# Supplementary material for: Protein restriction slows the development and progression of pathology in a mouse model of Alzheimer’s disease
Source: Nat Commun. 2024 Jun 18;15:5217. doi: 10.1038/s41467-024-49589-z (PMC11189507; doi:10.1038/s41467-024-49589-z)

Supplementary Figure 1

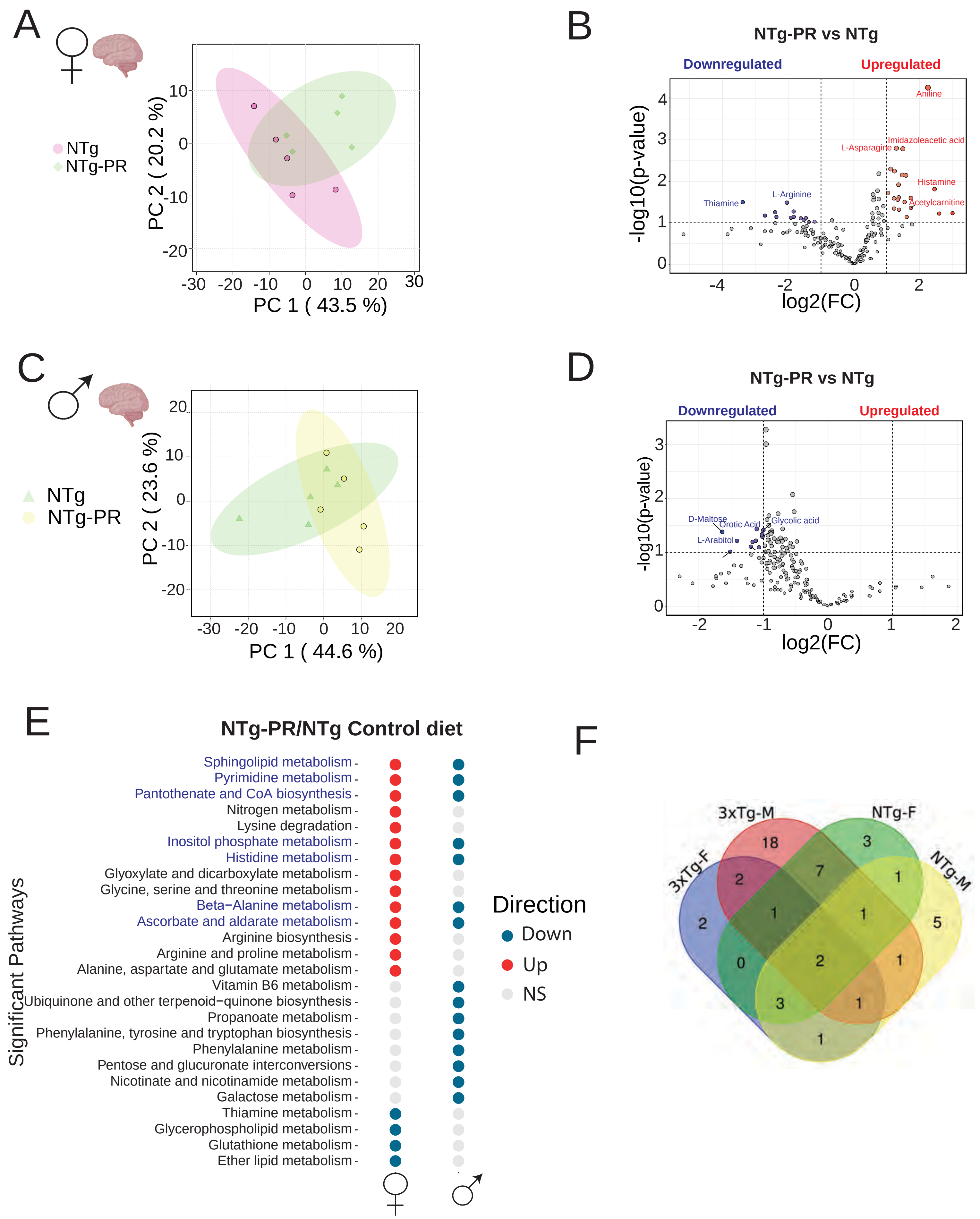

Supplementary Fig. 1: PR induced shifts in the brain metabolome of NTg mice. Untargeted metabolomics analysis was conducted on the whole brain of NTg female (A-B), and male (C-D) mice fed the indicated diets. (A, C) Principal Component Analysis (PCA) of brain metabolites from NTg females and males. (B, D) Volcano plots display altered brain metabolites, with blue and red indicating significantly decreased and increased metabolites between control and PR-fed NTg groups. Gray dots indicate metabolites with no significant difference (two tailed, t test, unadjusted P value < 0.05) with a > 2-fold change are labeled on the volcano plot. (E) Significantly up and down regulated pathways for each sex and diet were determined using metabolite set enrichment analysis (MSEA). n=5 biologically independent mice per group. Shared pathways between females and males are highlighted (F) Venn diagram illustrates shared metabolites altered in each genotype and sex of mouse by PR. Supplementary Fig. 1 brain icons in panels A and C created with BioRender.com, released under a Creative Commons Attribution-Non-Commercial-No Derivs 4.0 International license (Agreement number: BS26S7MR00). Source data are provided as a Source Data file.

Supplementary Figure 2

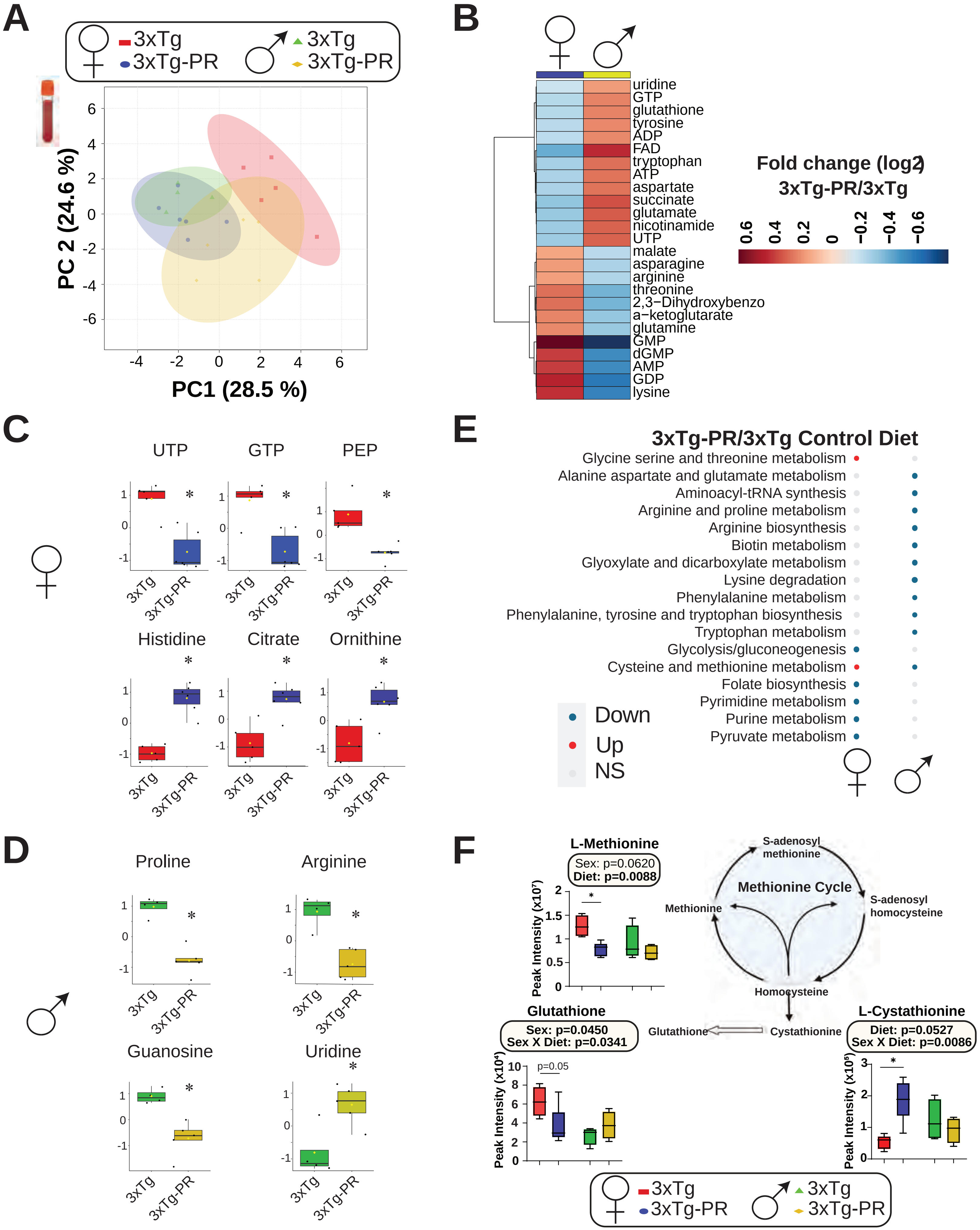

Supplementary Fig. 2: PR induces sex-specific shifts in the plasma metabolome of 3xTg mice. Targeted metabolomics analysis was conducted on the plasma of 3xTg female and male mice fed the indicated diets. (A) Principal Component Analysis (PCA) of plasma metabolites from 3xTg mice. (B) Heat map of the top 25 serum metabolites altered by PR feeding in 3xTg mice, represented as log2 fold change from Control-fed 3xTg mice of the same sex. (C-D) Plasma metabolites significantly altered by PR feeding in female (C) and male (D) 3xTg mice. (E) KEGG pathways significantly altered by PR in each sex. (F) Metabolites related to the methionine cycle and its intermediates. (A) For females n=5 Control-fed 3xTg, n=6 PR-fed 3xTg and for males n=4 Control-fed 3xTg and 5 PR-fed 3xTg biologically independent mice. (B) For females n=6 PR-fed 3xTg and for males n=5 PR-fed 3xTg biologically independent mice. (C) For females n=5 Control-fed 3xTg and n=6 PR-fed 3xTg biologically independent mice. (D) For males n=4 Control-fed 3xTg and n=5 PR-Fed 3xTg biologically independent mice. (E) For females n=6 PR-fed 3xTg and for males n=5 PR-fed 3xTg biologically independent mice. (F) For females n=5 Control-fed 3xTg, n=6 PR-fed 3xTg and for males n=4 Control-fed 3xTg and 4 PR-fed 3xTg biologically independent mice. Data from female control and PR fed 3xTg mice are plotted with red and blue bars respectively and data from male control and PR fed 3xTg mice are plotted with green and yellow bars (C-D) $*p<0.05$, two tailed t-test; (F) statistics for the overall effects of diet, sex, and the interaction represent the p value from a 2-way ANOVA; $*p<0.05$, from a Sidak's post-test examining the effect of parameters identified as significant in the 2-way ANOVA. Overlaid box plots show center as median and 25th-75th percentiles; whiskers represent minima and maxima. Data represented as mean ± SEM. Supplementary Fig. 2 plasma icon in panel A created with BioRender.com, released under a Creative Commons Attribution-Non-Commercial-No Derivs 4.0 International license (Agreement number: TV26S7N4OQ). Source data are provided as a Source Data file.

Supplementary Fig 3

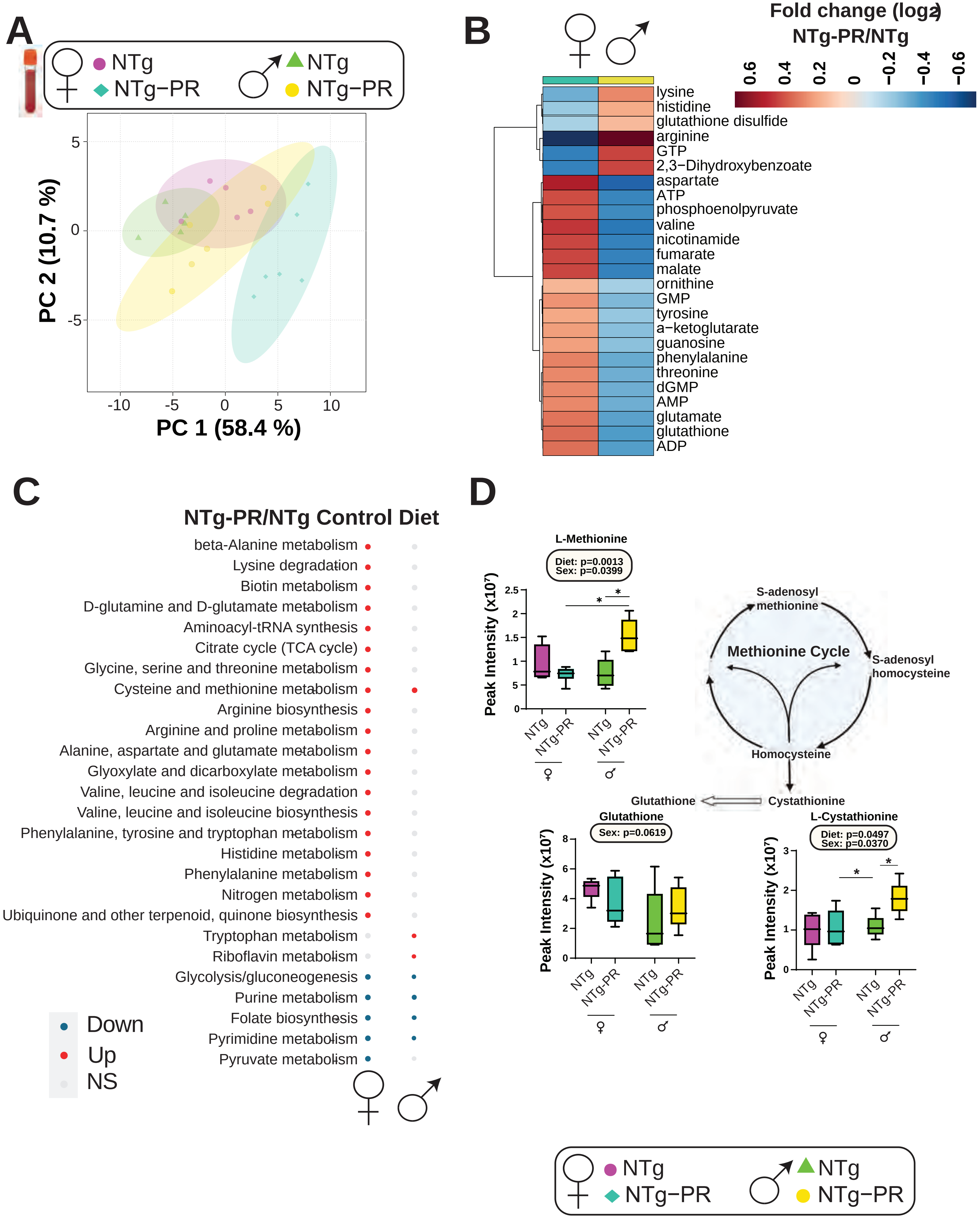

Supplementary Fig. 3: Distinct metabolic signatures in male and female NTg mice following long term PR. (A) Principal Component Analysis (PCA) of NTg males and females. (B) Heat map representation of the top 25 altered serum metabolites in both NTg-PR fed females and males, represented as log2 fold change from NTg mice on a control diet. (C) Significantly up and down regulated pathways for each sex and diet were determined using KEGG enrichment. (D) Methionine cycle and its intermediates are summarized. (A) For females n=5 Control-fed NTg, 6 PR-fed NTg and for males n=5 Control-fed NTg, 6 PR-fed NTg biologically independent mice. (B-C) For females n=6 PR-fed NTg and for males n=6 PR-fed NTg biologically independent mice. (D) For females n=5 Control-fed NTg, 6 PR-fed NTg and for males n=5 Control-fed NTg, 5 PR-fed NTg biologically independent mice. Data from female control and PR fed NTg mice are plotted with pink and bluish green bars respectively and data from male control and PR fed NTg mice are plotted with green and yellow bars. Statistics for the overall effects of diet, sex, and the interaction represent the p value from a 2-way ANOVA, *$p<0.05$, from a Sidak's post-test examining the effect of parameters identified as significant in the 2-way ANOVA. Data represented as mean ± SEM. Supplementary Fig. 3 plasma icon in panel A created with BioRender.com, released under a Creative Commons Attribution-Non-Commercial-No Derivs 4.0 International license (Agreement number: TV26S7N4OQ). Source data are provided as a Source Data file.

Supplementary Figure 4

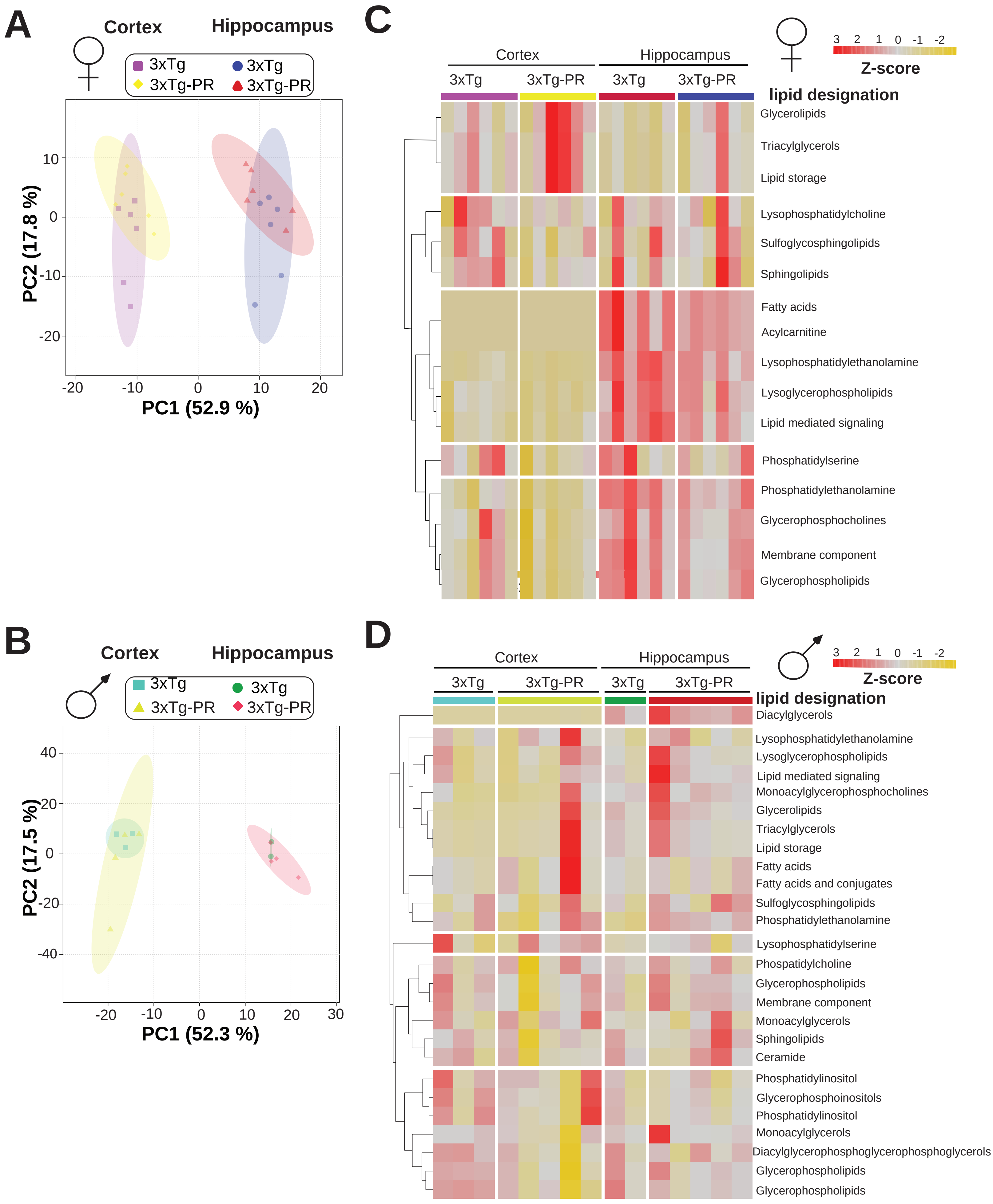

Supplementary Figure 4: Region specific lipidome changes in the brains of 3xTg mice.

(A-D) PCA of the variations in lipid classes in the hippocampus and cortex of female (A) and male(C) 3xTg mice. Heat maps of altered lipid classes in cortex and hippocampus of females (B) and males (D). (A-B) For females n=6 biologically independent mice/ group for both cortex and hippocampus (C-D) For males n=3 Control-fed, 5 PR-fed mice for cortex and n=2 Control-fed, and 5 PR-fed for hippocampus biologically independent mice were used. Source data are provided as a Source Data file.

Supplementary Figure 5

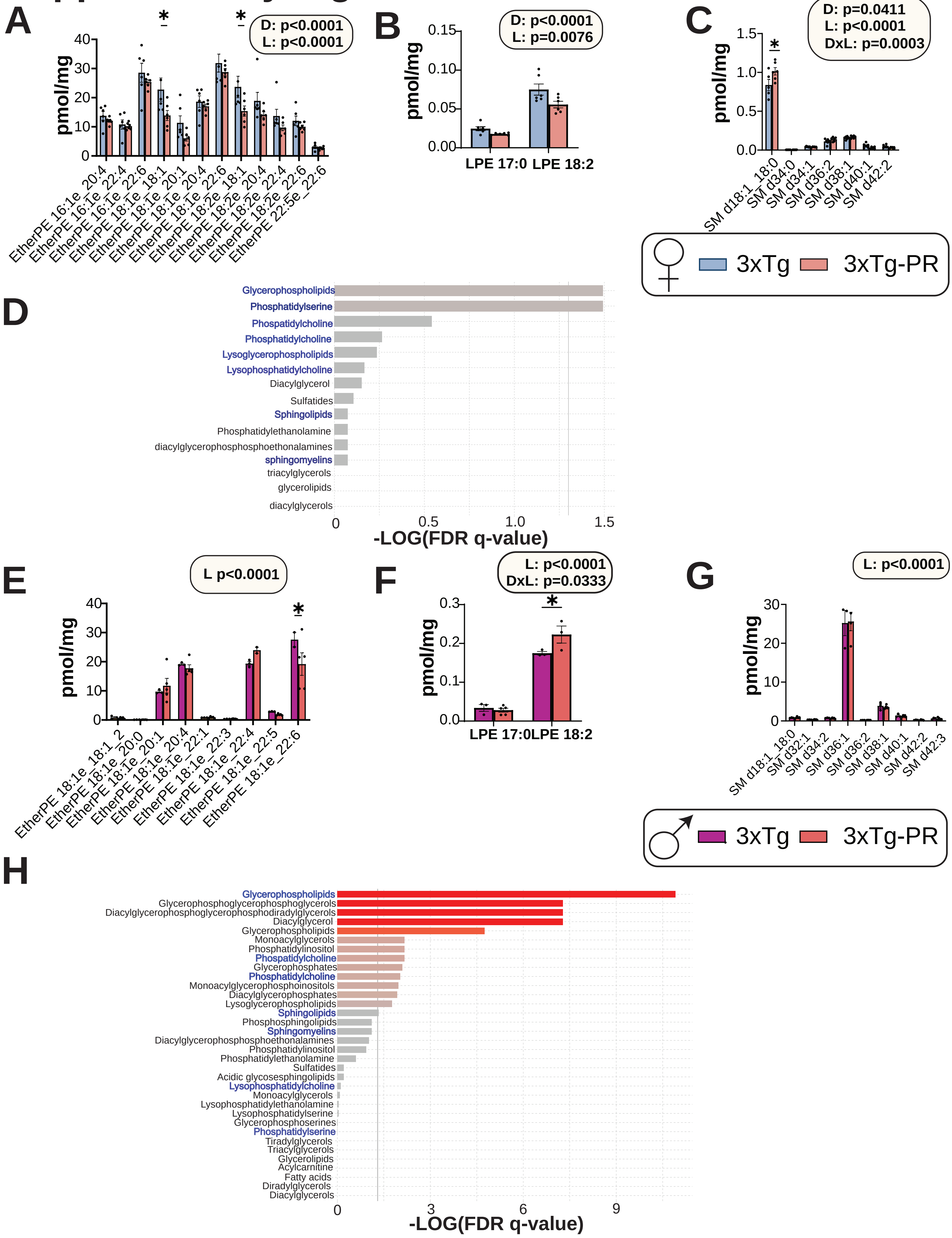

Supplementary Figure 5: PR induces sex-specific shifts in the brain lipidome of 3xTg mice.

(A-H) Lipidomic analysis was performed on the cortex of female (A-D) and male (E-H) 3xTg mice fed either a Control or PR diet. (D, H) LION ontology pathway enrichment analysis of the complete lipidomic dataset from the cortex of female (D) and male (H) 3xTg mice. The vertical gray lines indicate the threshold value for significant enrichments; names in blue are pathways enriched in both sexes. (A-D) For females n=6 Control-fed 3xTg and 6 PR-fed 3xTg biologically independent mice. (E-H) For males n=3 Control-fed 3xTg and 5 PR-fed 3xTg biologically independent mice. statistics for the overall effects of diet, lipid and the interaction represent the p value from a 2-way ANOVA; *$p<0.05$, from a Sidak's post-test for the effect of PR on each lipid. Data from female control and PR fed 3xTg mice are plotted with grey and blue bars respectively and data from male control and PR fed 3xTg mice are plotted with fuchsia pink and coral pink bars. Ether PE: Ether phosphatidylethanolamines; LPE: lyso-phosphatidylethanolamines; SM: sphingomyelin. Data represented as mean ± SEM. Source data is provided as a Source Data file.

Supplementary Figure 6

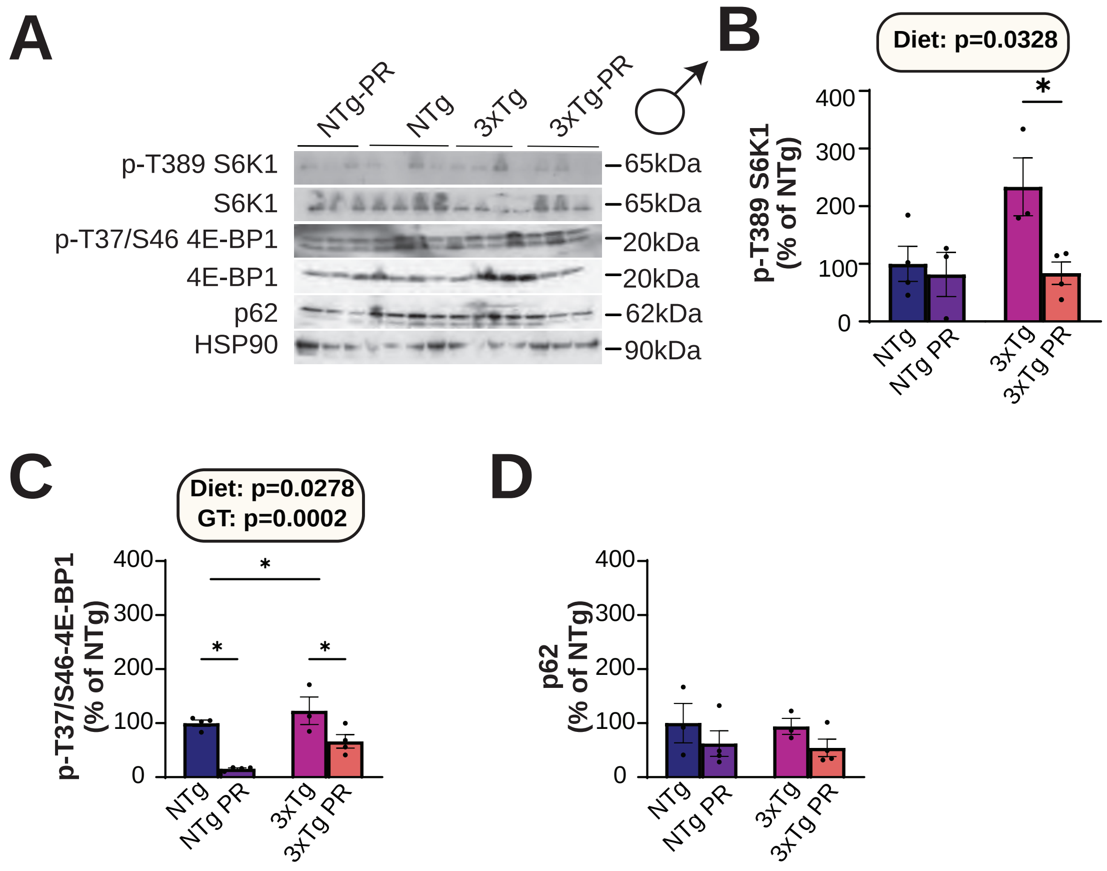

Supplementary Figure 6: PR reduces mTORC1 signaling in the brains of male 3xTg mice. Representative immunoblots of the whole brain of male mice (A). Quantification of the mTORC1 substrates T389 S6K1 (B) and T37/S46 4E-BP1 (C). (D) Quantification of p62 expression. (B-D) n=4 Control-fed NTg, 3 PR-fed NTg, 3 Control-fed 3xTg and 4 PR-fed 3xTg biologically independent mice; statistics for the overall effects of genotype (GT), diet, and the interaction represent the p value from a 2-way ANOVA, *$p<0.05$, from a Sidak's post-test examining the effect of parameters identified as significant in the 2-way ANOVA. Data from male control and PR fed NTg mice are plotted with blue and purple bars respectively and data from control and PR fed 3xTg mice are plotted with fuchsia pink and coral pink bars. Data represented as mean ± SEM. Source data is provided as a Source Data file.

Supplementary Figure 7

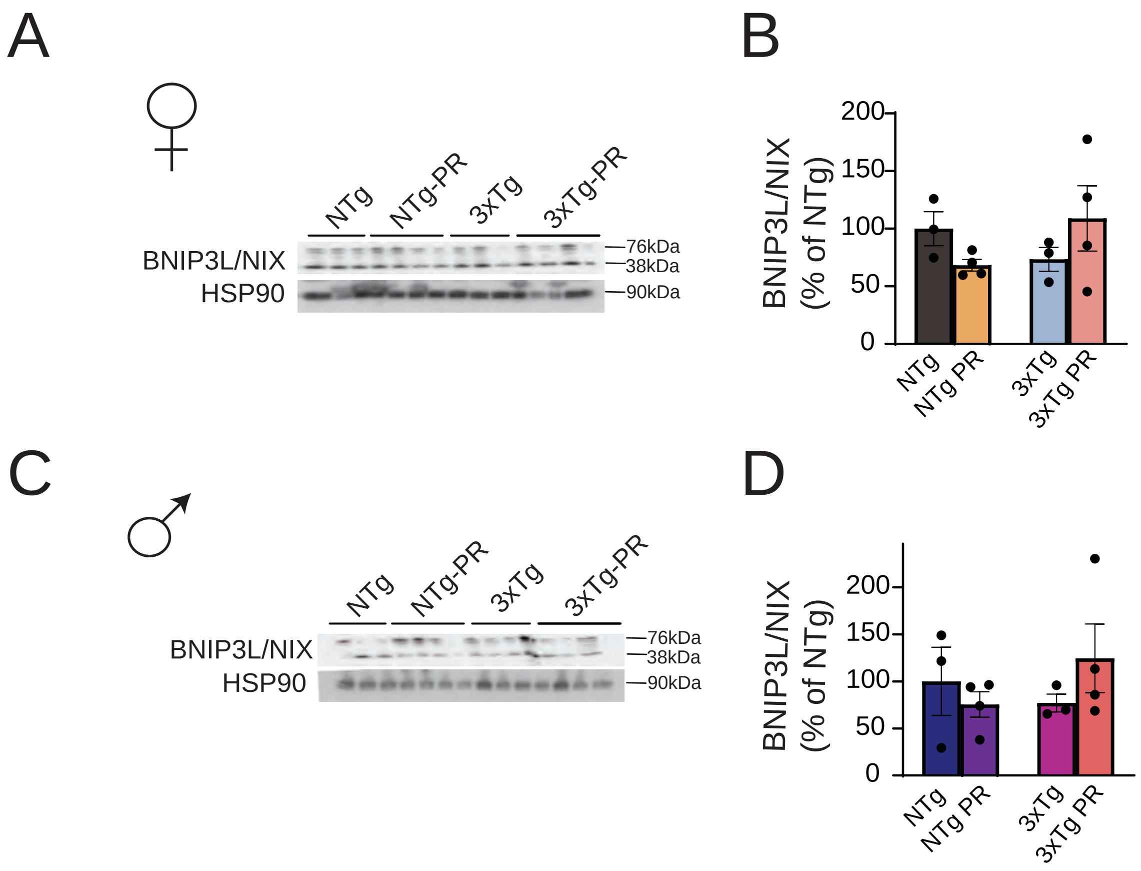

Supplementary Figure 7: Effect of PR on mitophagy in the brains of 3xTg mice. Representative immunoblots of BNIP3L/NIX expression in the whole brain of female and male mice (A, C). Quantification of BNIP3L/NIX expression in female and male mice (B, D). (B-D) n=3 Control-fed NTg, 4 PR-fed NTg, 3 Control-fed 3xTg and 4 PR-fed 3xTg biologically independent mice; a 2-way ANOVA for the overall effects of genotype (GT), diet, and the interaction examined. Data from female control and PR fed NTg mice are plotted with grey and yellow bars respectively and data from control and PR fed 3xTg mice are plotted with blue and pink bars. Data from male control and PR fed NTg mice are plotted with blue and purple bars respectively and data from control and PR fed 3xTg mice are plotted with fuchsia pink and coral pink bars. Source data are provided as a Source Data file.

Supplementary Figure 8

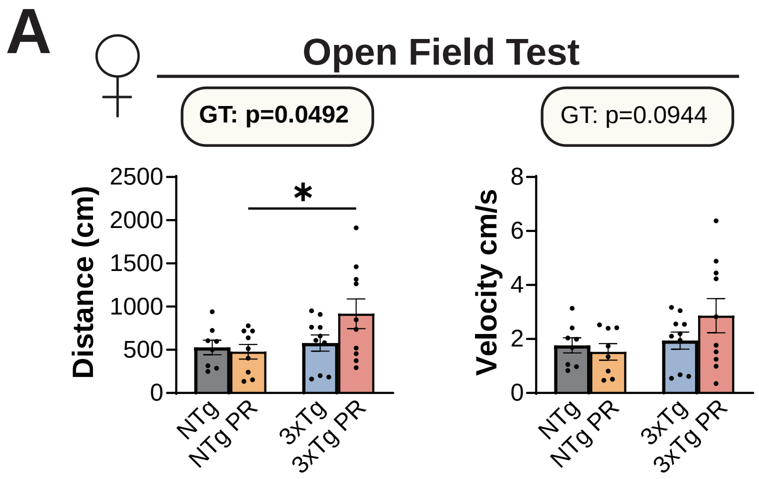

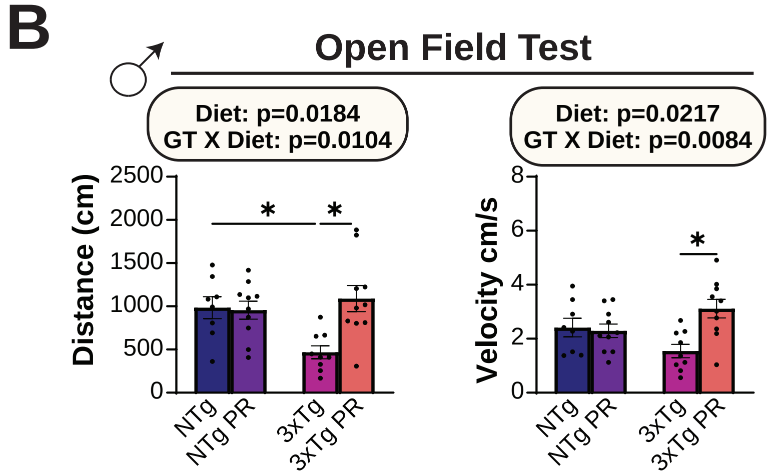

Supplementary Figure 8: Effect of PR on open field test performance. (A-B) Distance and velocity of female (A) and male (B) mice during open field test. (A) For females n=8 Control-fed NTg, 9 PR-fed NTg, 10 Control-fed 3xTg and 10 PR-fed 3xTg biologically independent mice. (B) n=8 Control-fed NTg, 10 PR-fed NTg, 10 Control-fed 3xTg and 10 PR-fed 3xTg biologically independent mice; statistics for the overall effects of genotype (GT), diet, and the interaction represent the p value from a 2-way ANOVA, *$p<0.05$, from a Sidak's post-test examining the effect of parameters identified as significant in the 2-way ANOVA. Data from female control and PR fed NTg mice are plotted with grey and yellow bars respectively and data from control and PR fed 3xTg mice are plotted with blue and pink bars. Data from male control and PR fed NTg mice are plotted with blue and purple bars respectively and data from control and PR fed 3xTg mice are plotted with fuchsia pink and coral pink bars. Data represented as mean ± SEM. Source data is provided as a Source Data file.

Females

Figure 6

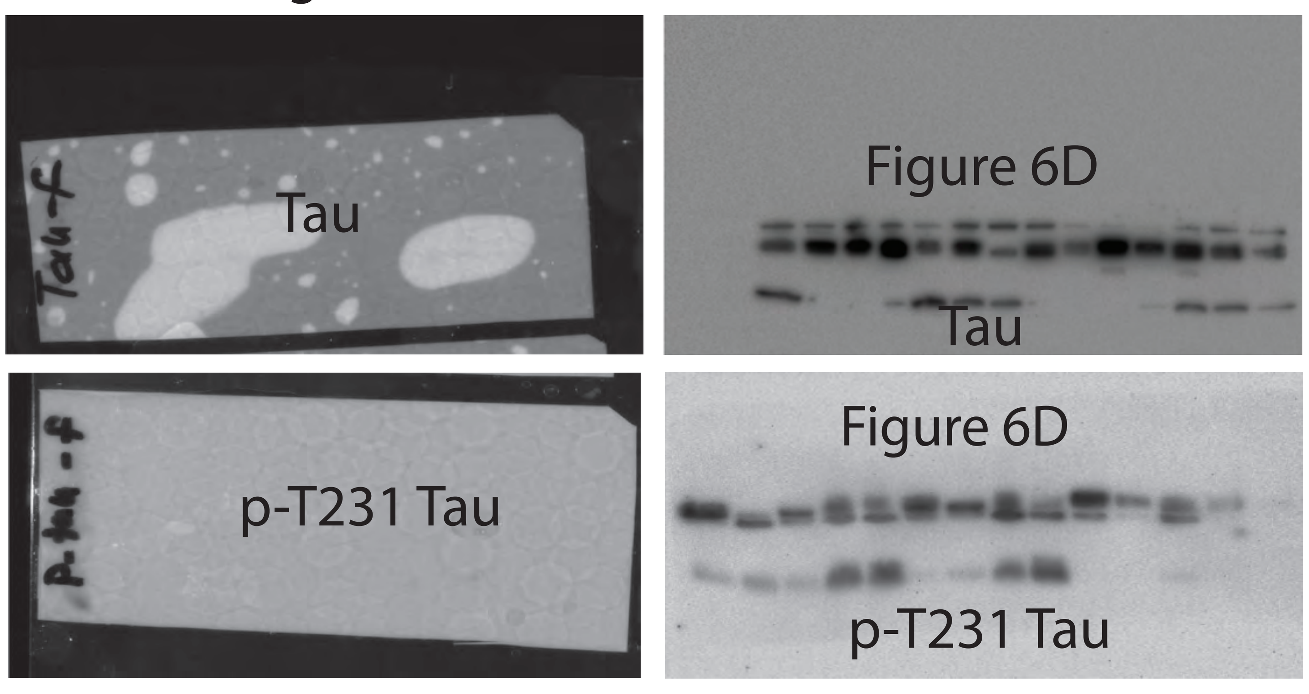

Figure 7

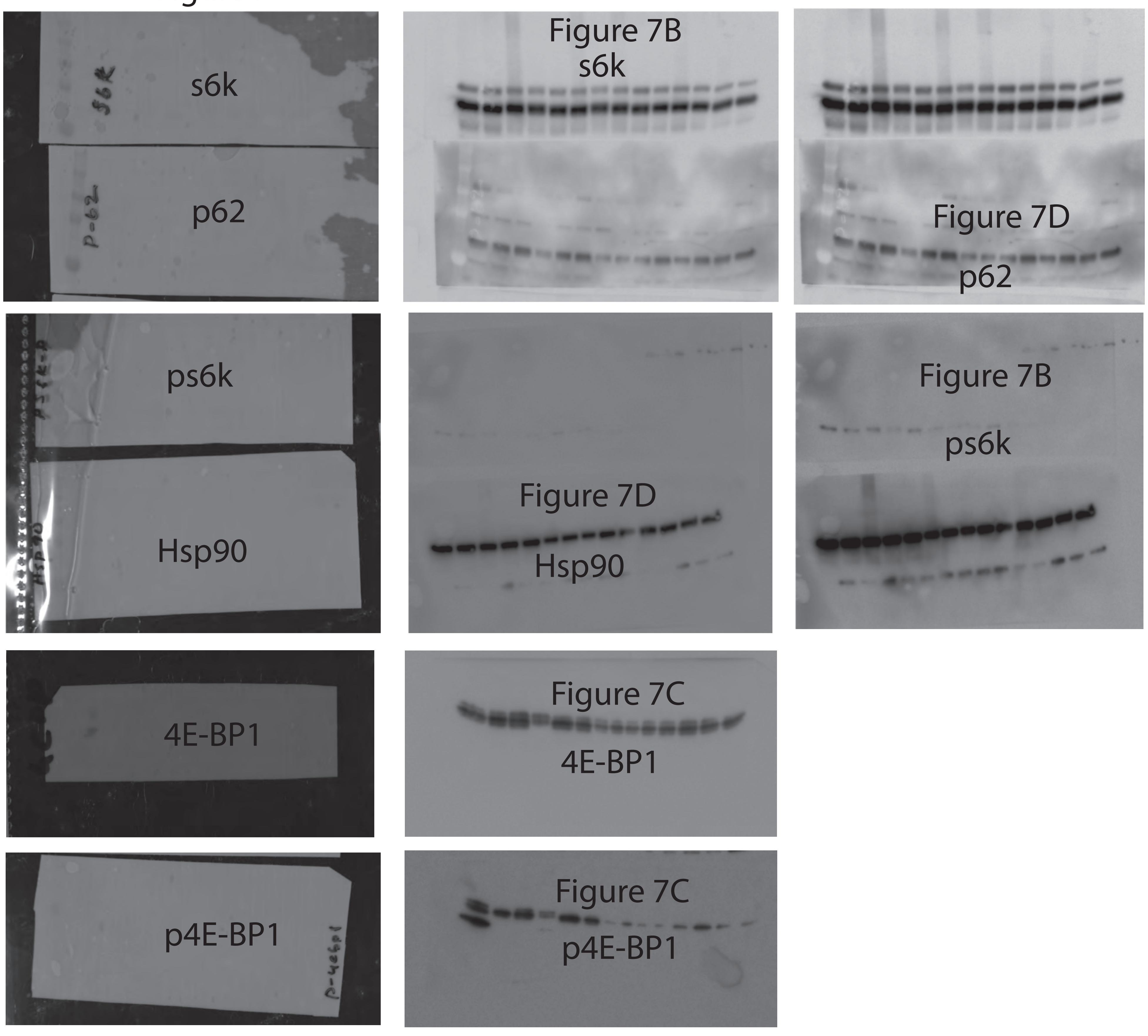

Males

Figure 6A

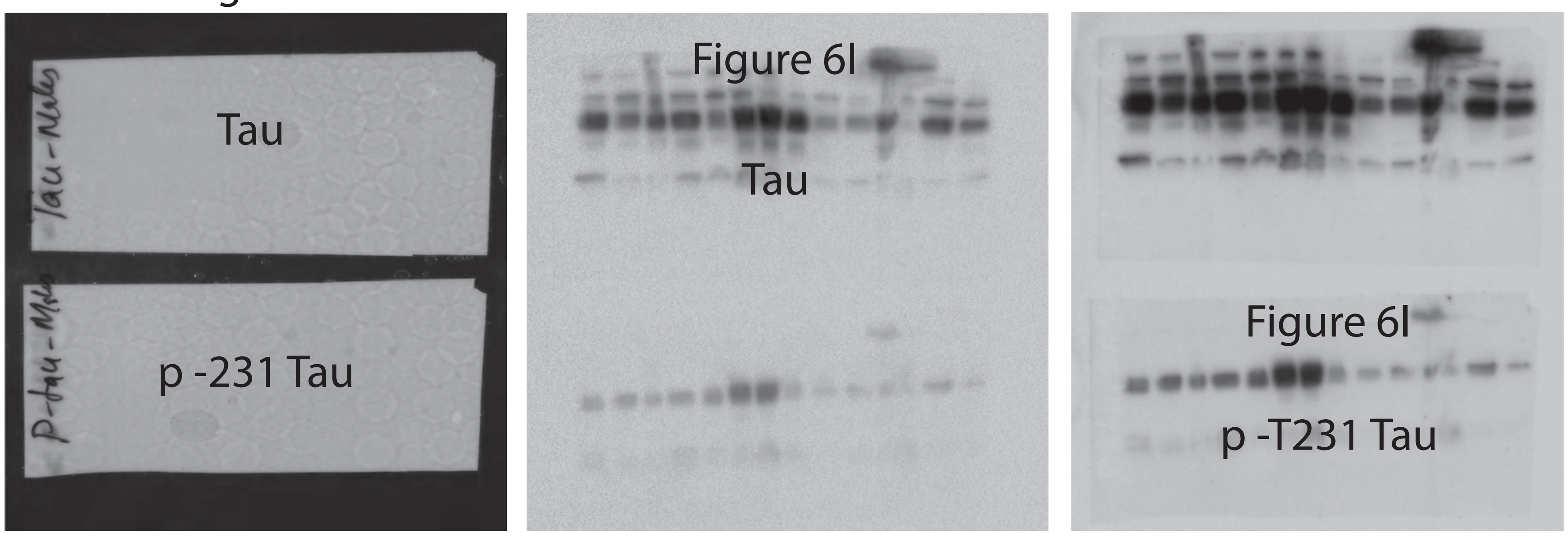

Supplementary Figure 6A

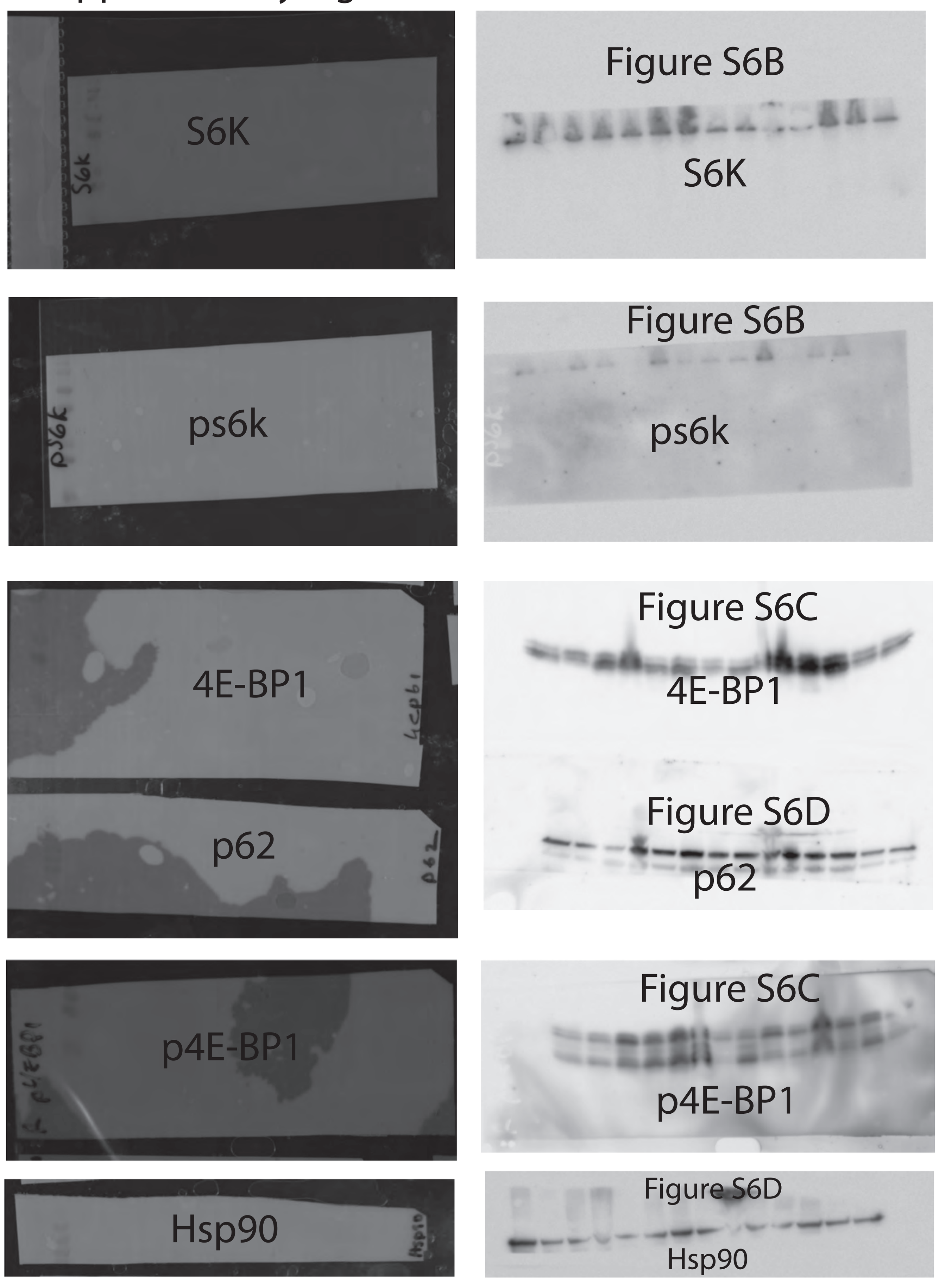

Supplementary Figure 7

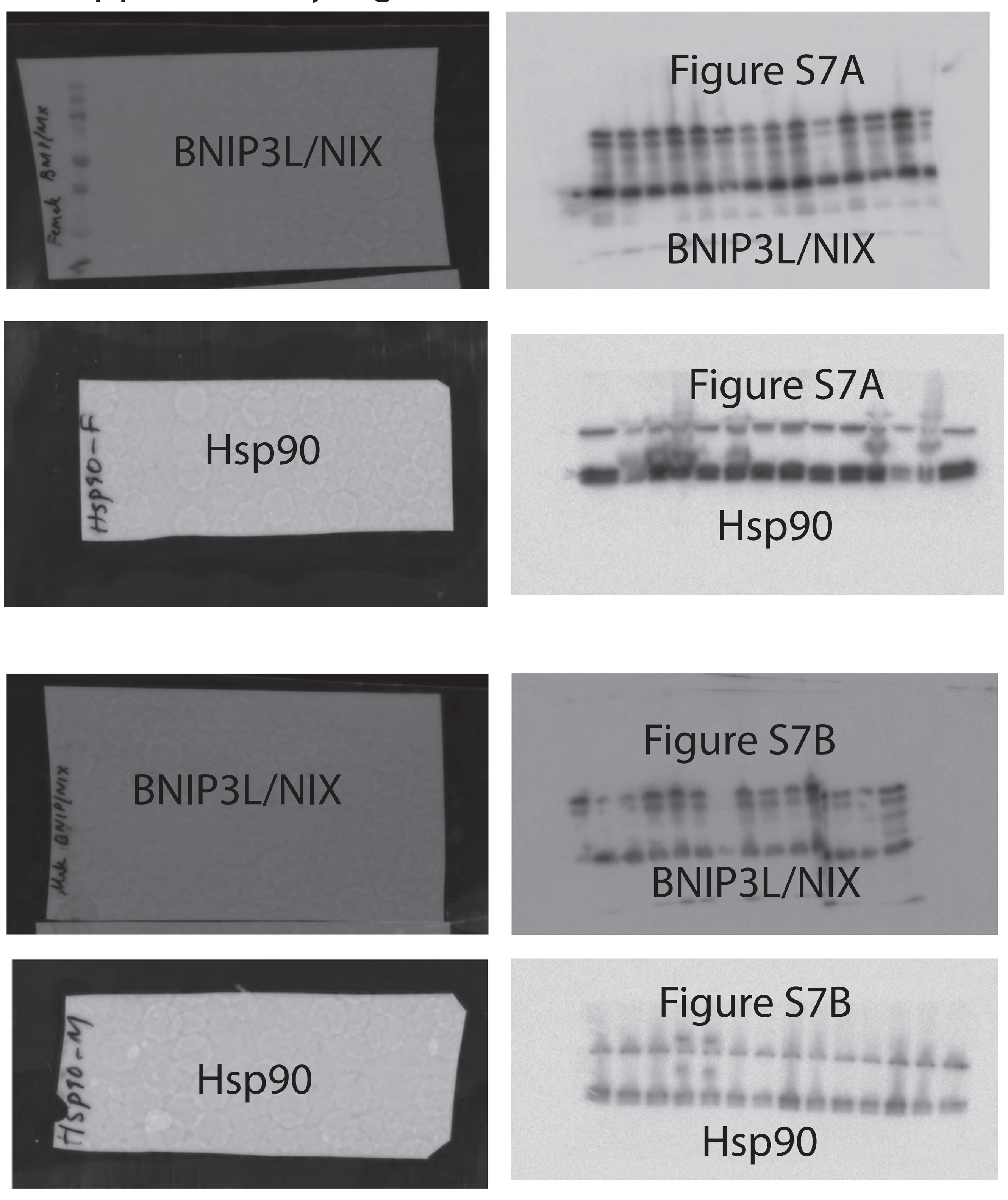

Supplement: Supplementary file 1 — Supplementary Information [file 41467_2024_49589_MOESM1_ESM.pdf]
