# Supplementary material for: Protein restriction slows the development and progression of pathology in a mouse model of Alzheimer’s disease
Source: Nat Commun. 2024 Jun 18;15:5217. doi: 10.1038/s41467-024-49589-z (PMC11189507; doi:10.1038/s41467-024-49589-z)
Supplement: Supplementary file 3 — Description of Additional Supplementary Files [file 41467_2024_49589_MOESM3_ESM.pdf]

Description of Additional Supplementary Files

Supplementary Data 1: Diet composition and calorie content for diets used in this study.

Supplementary Data 2: List of all 194 metabolites and their compound names identified through untargeted metabolomics in the brain.

Supplementary Data 3: Significantly altered brain metabolites identified in both female and male 3xTg mice. Two-tailed t test, unadjusted P value < 0.05 with a > 2-fold change are labeled on the volcano plot.

Supplementary Data 4: Significantly altered brain metabolites identified in both female and male NTg mice. Two-tailed t test, unadjusted P value < 0.05 with a > 2-fold change are labeled on the volcano plot.

Supplementary Data 5: Pathway enrichment analysis of the brain metabolites in 3xTg mice. Significantly up and down regulated pathways for each sex and diet were determined using metabolite set enrichment analysis (MSEA) unadjusted P value < 0.1.

Supplementary Data 6: Pathway enrichment analysis of the brain metabolites in NTg mice. Significantly up and down regulated pathways for each sex and diet were determined using metabolite set enrichment analysis (MSEA) unadjusted P value < 0.1.

Supplementary Data 7: List of all 38 metabolites and their compound names identified through metabolomics in the plasma.

Supplementary Data 8: Log_2 fold-changes from plasma metabolomic analysis in 3xTg and NTg females and males found in **Supplementary Figure 2B and 3B**.

Supplementary Data 9: Results output from pathway analysis in the plasma which shows pathway direction and p-values in both females and males. Significantly up and down regulated pathways for each sex and diet were determined using metabolite set enrichment analysis (MSEA) unadjusted P value < 0.05.

Supplementary Data 10: Untargeted lipidomics data from mass spectrometry analysis of hippocampus and cortex in females.

Supplementary Data 11: Untargeted lipidomics data from mass spectrometry analysis of hippocampus and cortex in males

Supplementary Data 12: Results of LION lipid ontology analysis in female 3xTg mice. Lipid ontology (LION) pathway enrichment of significantly altered lipids $p < 0.05$.

Supplementary Data 13: Results of LION lipid ontology analysis in male 3xTg mice. Lipid ontology (LION) pathway enrichment of significantly altered lipids $p < 0.05$.

Supplementary Data 14: Targeted brain lipidomics data from male and female NTg and 3xTg mice

Supplementary Data 15: Antibodies used for both western blotting and immunohistochemistry.
