# Supplementary material for: Protein restriction slows the development and progression of pathology in a mouse model of Alzheimer’s disease
Source: Nat Commun. 2024 Jun 18;15:5217. doi: 10.1038/s41467-024-49589-z (PMC11189507; doi:10.1038/s41467-024-49589-z)
Supplement: Supplementary file 5 — Reporting Summary [file 41467_2024_49589_MOESM5_ESM.pdf]

nature portfolio

Corresponding author(s): Dudley Lamming

Last updated by author(s): May 20, 2024

Reporting Summary

Nature Portfolio wishes to improve the reproducibility of the work that we publish. This form provides structure for consistency and transparency in reporting. For further information on Nature Portfolio policies, see our Editorial Policies and the Editorial Policy Checklist.

Statistics

For all statistical analyses, confirm that the following items are present in the figure legend, table legend, main text, or Methods section.

n/a	Confirmed	
☐	☒	The exact sample size (*n*) for each experimental group/condition, given as a discrete number and unit of measurement
☐	☒	A statement on whether measurements were taken from distinct samples or whether the same sample was measured repeatedly
☐	☒	The statistical test(s) used AND whether they are one- or two-sided<br>*Only common tests should be described solely by name; describe more complex techniques in the Methods section.*
☐	☒	A description of all covariates tested
☐	☒	A description of any assumptions or corrections, such as tests of normality and adjustment for multiple comparisons
☐	☒	A full description of the statistical parameters including central tendency (e.g. means) or other basic estimates (e.g. regression coefficient) AND variation (e.g. standard deviation) or associated estimates of uncertainty (e.g. confidence intervals)
☐	☒	For null hypothesis testing, the test statistic (e.g. *F*, *t*, *r*) with confidence intervals, effect sizes, degrees of freedom and *P* value noted<br>*Give P values as exact values whenever suitable.*
☒	☐	For Bayesian analysis, information on the choice of priors and Markov chain Monte Carlo settings
☒	☐	For hierarchical and complex designs, identification of the appropriate level for tests and full reporting of outcomes
☒	☐	Estimates of effect sizes (e.g. Cohen's *d*, Pearson's *r*), indicating how they were calculated

Our web collection on statistics for biologists contains articles on many of the points above.

Software and code

Policy information about availability of computer code

Data collection	Used softwares for metabolic chambers (Oxymax v. 5.64 software from Columbus Instruments) and behavioral phenotyping (EthoVision XT) from Noldus.
Data analysis	Data in this paper was analysed using Office 365, Graphpad Prism 9, LION Lipid Ontology, MetaboAnalyst 5.0 and NIH Image J 1.53t

For manuscripts utilizing custom algorithms or software that are central to the research but not yet described in published literature, software must be made available to editors and reviewers. We strongly encourage code deposition in a community repository (e.g. GitHub). See the Nature Portfolio guidelines for submitting code & software for further information.

Data

Policy information about availability of data

All manuscripts must include a data availability statement. This statement should provide the following information, where applicable:

- Accession codes, unique identifiers, or web links for publicly available datasets
- A description of any restrictions on data availability
- For clinical datasets or third party data, please ensure that the statement adheres to our policy

This manuscript includes data availability statement with the necessary information on metabolomics and lipidomics data deposition. The data that support the plots and other findings of this study are provided as a Source Data file.

Research involving human participants, their data, or biological material

Policy information about studies with human participants or human data. See also policy information about sex, gender (identity/presentation), and sexual orientation and race, ethnicity and racism.

Reporting on sex and gender	*Use the terms sex (biological attribute) and gender (shaped by social and cultural circumstances) carefully in order to avoid confusing both terms. Indicate if findings apply to only one sex or gender; describe whether sex and gender were considered in study design; whether sex and/or gender was determined based on self-reporting or assigned and methods used. Provide in the source data disaggregated sex and gender data, where this information has been collected, and if consent has been obtained for sharing of individual-level data; provide overall numbers in this Reporting Summary. Please state if this information has not been collected. Report sex- and gender-based analyses where performed, justify reasons for lack of sex- and gender-based analysis.*
Reporting on race, ethnicity, or other socially relevant groupings	*Please specify the socially constructed or socially relevant categorization variable(s) used in your manuscript and explain why they were used. Please note that such variables should not be used as proxies for other socially constructed/relevant variables (for example, race or ethnicity should not be used as a proxy for socioeconomic status). Provide clear definitions of the relevant terms used, how they were provided (by the participants/respondents, the researchers, or third parties), and the method(s) used to classify people into the different categories (e.g. self-report, census or administrative data, social media data, etc.) Please provide details about how you controlled for confounding variables in your analyses.*
Population characteristics	*Describe the covariate-relevant population characteristics of the human research participants (e.g. age, genotypic information, past and current diagnosis and treatment categories). If you filled out the behavioural & social sciences study design questions and have nothing to add here, write "See above."*
Recruitment	*Describe how participants were recruited. Outline any potential self-selection bias or other biases that may be present and how these are likely to impact results.*
Ethics oversight	*Identify the organization(s) that approved the study protocol.*

Note that full information on the approval of the study protocol must also be provided in the manuscript.

Field-specific reporting

Please select the one below that is the best fit for your research. If you are not sure, read the appropriate sections before making your selection.

☒ Life sciences ☐ Behavioural & social sciences ☐ Ecological, evolutionary & environmental sciences

For a reference copy of the document with all sections, see nature.com/documents/nr-reporting-summary-flat.pdf

Life sciences study design

All studies must disclose on these points even when the disclosure is negative.

Sample size	Sample sizes for all the metabolic studies were determined based on our lab's previously published experimental results using various dietary interventions (Fontana et al, 2016, Cell Reports, Green et al, 2022, Cell Metabolism), with the goal of having > 90% power to detect a change in area under the curve during a GTT ($p<0.05$). Sample sizes for behavioral studies were chosen based on the guidelines for studies in aging animals reported by the MouseAGE consortium (I Bellantuono et al, 2020, Nature Protocols). Sample sizes for molecular analyses and histopathology were chosen in consultation with AD researchers and their recent studies (Rigby, M. J, 2021, Commun Biol).
Data exclusions	Outliers were excluded based on Grubbs' test, also known as the ESD method (extreme studentized deviate).
Replication	We chose the assays based upon evaluation of a number of criteria including good reproducibility, and they are widely utilized by multiple groups. The Control and Low Protein diets successfully reproduce results found in our previous studies as well as those from other laboratories. All data derived from animal experiments represent the results obtained from at least three biological independent animals. As these were long-term longitudinal studies, there were no formal attempts at replication, but the metabolic and behavioral effects of the Low Protein diet observed in an initial cohort of male mice were replicated in a larger followup study. Molecular Analysis and histopathology were done once following the end of experiments (Rigby, M. J, 2021, Commun Biol).
Randomization	Prior to the beginning of in vivo studies, the animals of each sex and strain were randomized at the cage level into groups of equivalent weight.
Blinding	Investigators were blinded to diet groups during metabolic and behavioral data collection whenever feasible, but this was not usually possible or feasible as all the cages were clearly marked to indicate the diet provided, diets were color-coded to prevent feeding mistakes and the strain, sex, size and body composition of the mice was altered by genotype and diet. Necropsies were not blinded either as the diet and strain differences were easily distinguishable. During histology analysis and interpretation, investigators were blinded whenever feasible. Blinding is not relevant to the majority of the studies conducted here, as the data is collected in numeric form which is not readily subject to bias due to the need for subjective interpretation; this includes behavioral data, as the extraction and generation of data were mostly automatically generated by the software from EthoVIsion.

Reporting for specific materials, systems and methods

We require information from authors about some types of materials, experimental systems and methods used in many studies. Here, indicate whether each material, system or method listed is relevant to your study. If you are not sure if a list item applies to your research, read the appropriate section before selecting a response.

Materials & experimental systems

n/a	Involved in the study
☐	☒ Antibodies
☒	☐ Eukaryotic cell lines
☒	☐ Palaeontology and archaeology
☐	☒ Animals and other organisms
☒	☐ Clinical data
☒	☐ Dual use research of concern
☒	☐ Plants

Methods

n/a	Involved in the study
☒	☐ ChIP-seq
☒	☐ Flow cytometry
☒	☐ MRI-based neuroimaging

Antibodies

Antibodies used

Primary Antibodies : S6K (1:1,000; #2708S), p-p70 S6K1 (!:500 #9234L), p-T37/S46 4E-BP1 (1:1000; #2855), 4E-BP1 (1:1000; #9452), HSP90 (1:1000; #4877) and BNIP3L/Nix (1:1000;#12396) were purchased from Cell Signaling Technologies (CST, Danvers, MA, USA). Antibodies to Phospho Tau AT180 (1:1000; #MN1040), Tau HT7 (1:1000; #MN1000), anti-GFAP (1:1000; PIMA512023), were purchased from Thermo Fisher Scientifc (Thermo Fisher, Waltham, MA, USA). Antibodies to SQSTM1/p62 (1:1000; #03-GP62-C), was purchased from American Research products (ARP, Waltham, MA, USA). Antibody to anti-IBA1 (1:1000; #ab178847) was purchased from Abcam (Abcam, Waltham, MA, USA)

Secondary Antibodies: Anti rabbit secondary antibody (1:2000; #7074) was purchased from Cell Signalling Technology (CST, Danvers, MA, USA), Alexa 647 donkey anti-chicken (1:200; #NC0494952), Alexa 546 goat anti-rabbit (1:200; #A-11035), Alexa 488 goat anti-mouse (1:200, #A28175) were purchased from Thermo fisher scientific (Thermo Fisher, Waltham, MA, USA). Guinea pig secondary antibody (90001) was purchased from American Research products (ARP, Waltham, MA, USA).

Stain: Thioflavin-S (T-3156) was purchased from Sigma Aldrich (Sigma, St, Louis, Mo, USA)

Validation

All antibodies were well validated for use in mice by the manufacturer and validation descriptions are available on the
CST website https://www.cellsignal.com.
Thermo fisher website, https://www.thermofisher.com/us/en/home.html.
American Research products website, https://www.arp1.com/.
Abcam https://www.abcam.com/ .
Specificity of antibodies for molecular as well as histology has also been validated by the authors in previous publications, including Lamming et al, 2014, Aging Cell; Baar et al, 2016, Aging Cell, Rigby, M. J, 2021, Commun Biol.

Animals and other research organisms

Policy information about studies involving animals; ARRIVE guidelines recommended for reporting animal research, and Sex and Gender in Research

Laboratory animals

Six month old male and female homozygous 3xTg-AD mice and non-transgenic controls were used; these animals were bred at our animal facility in Madison from stock obtained from JAX-MMRC (MMRRC Item Number 034830) and The Jackson Laboratory (strain 101045).

Wild animals

No wild animals were used in the study.

Reporting on sex

We used both male and female mice in this study.

Field-collected samples

No field collected samples were used in the study.

Ethics oversight

Animal studies were approved by the Institutional Animal Care and Use Committee of the William S. Middleton Memorial Veterans Hospital, Madison USA.

Note that full information on the approval of the study protocol must also be provided in the manuscript.
